# The predictive value of neurally adjusted ventilatory assist indexes for the prognosis of patients with severe cerebral hemorrhage

**DOI:** 10.1186/s40001-023-01601-w

**Published:** 2024-01-03

**Authors:** Lin Yao, Zihao Zhou, Tao Yu, Guiliang Wang, Zhen Fan, Yun Tang

**Affiliations:** 1 Department of Neurosurgery, The First Affiliated Hospital of Wannan Medical College (Yijishan Hospital of Wannan Medical College), Wuhu, 241001 People’s Republic of China; 2 The Translational Research Institute for Neurological Disorders of Wannan Medical College, Wuhu, 241001 People’s Republic of China

**Keywords:** Cerebral hemorrhage, Electrical activity of the diaphragm, Neuro-ventilatory efficiency, Neuro-muscular efficiency, Prognosis

## Abstract

**Objective:**

This study assessed the predictive value of electrical activity of the diaphragm (EAdi) and the EAdi-derived monitoring index in the prognosis of patients with severe cerebral hemorrhage.

**Methods:**

Ninety patients with severe cerebral hemorrhage were admitted to the Neurosurgery Intensive Care Unit of Yijishan Hospital from April 2019 to June 2021 and were divided into the good prognosis group (Glasgow Outcome Scale [GOS] ≥ 4) and poor prognosis group (GOS ≤ 3). The receiver operating characteristic (ROC) curve and area under the curve (AUC) were used to evaluate prediction accuracy.

**Results:**

EAdi, neuro-ventilatory efficiency (NVE), and neuro-muscular efficiency (NME) in patients with good prognosis were significantly higher than those in patients with poor prognosis (4.707 µV vs 2.80 µV, *P* < 0.001; 141.85 ml/µV vs 66.01 ml/µV, *P* = 0.000; 2.57 cm H_2_O/µV vs 1.37 cm H_2_O/µV, *P* = 0.000). The area under the ROC curve for the EAdi score was 0.719, with sensitivity of 69.70% and specificity of 68.42% when EAdi was 3.6 µV. The AUC for NVE score was 0.793, with sensitivity of 75.76% and specificity of 75.44% when the NVE value was 95.32 ml/µV. The AUC for NME score was 0.792, with sensitivity of 69.70% and specificity of 78.95% when the NME value was 2.06 H_2_O/µV. The 6-month survival time of patients with higher EAdi, NVE, and NME was significantly longer than that of patients with lower EAdi, NVE, and NME

**Conclusion:**

EAdi, NVE, and NME can be used as indices for predicting the prognosis of patients with severe cerebral hemorrhage.

*Trial registration* No.ChiCTR1900022861. Registered April 28, 2019, http://www.chictr.org.cn.

## Introduction

Acute spontaneous cerebral hemorrhage is the most common type of intracerebral hemorrhage, with an incidence of 60–80/10 million, accounting for 20–30% of all strokes, the overall prognosis of cerebral hemorrhage is poor, and the acute mortality rate can reach 30–40% [1]. The main causes of cerebral hemorrhage include intracranial sclerosis and rupture of blood vessels, intracranial vascular malformations, and rare causes include hematological diseases [2]. Patients with cerebral hemorrhage often have hypoxemia due to incomplete obstruction of the respiratory tract, hypoventilation, aspiration, and pulmonary infection [3]. Patients with severe cerebrovascular disease have a high incidence of impaired consciousness, severely impaired brainstem function, decreased airway motor function, and weakened or disappeared protective reflexes. These patients are more likely to have airway obstruction and aspiration symptoms, leading to respiratory failure. Mechanical ventilation is usually used to protect the airways from the risk of inhalation and prevent hypoxemia and hypercapnia [4, 5].

Neurally adjusted ventilatory assist (NAVA) is a new mode of mechanical ventilation that provides ventilation assistance in proportion to EAdi [6–8]. The NAVA is of little value if the patient's central respiratory system is completely disabled. NAVA can monitor and measure EAdi and is a relatively simple, minimally invasive tool for real-time monitoring of patient–ventilator interactions [9]. The unique feature of NAVA is that it can recognize the beginning of nerve expiration, whereas auxiliary control ventilation or pressure support ventilation (PSV) cannot. NAVA has more clinical implications, such as better patient–ventilator synchronization and more natural breathing patterns, which improve comfort and oxygenation. The EAdi allows quantification of the neural respiratory drive to the electrical activity of the diaphragm during pressure [10]. On the other hand, NAVA can be used as a monitoring tool to observe the functional changes to the respiratory center and its conduction pathway. However, this requires further research on a larger scale. EAdi is the sum of the action potentials of the diaphragmatic muscle fibers, which is related to the overall inspiratory effort of healthy subjects and patients with respiratory failure. When the respiratory central drive increases due to an increase in respiratory load, respiratory muscle weakness, or violent activity, the central nervous system will raise more muscle fibers by increasing the frequency of impulses and the number of nerve fibers transmitting impulses. In contrast, when the respiratory load decreases, the respiratory center has been shown to decrease the frequency of impulses and the number of nerve fibers transmitting impulses, and EAdi decreases. Therefore, it provides a reliable estimate of the central respiratory driving force [11, 12].

The EAdi and EAdi-derived monitoring index can be used as a powerful monitoring tool for any other respiratory mode, providing a large amount of valuable clinical information such as the patient's respiratory drive, ventilation demand, and the impact of ventilator settings on the patient, as well as judging the depth of sedation and the possibility of weaning. Because research on NAVA mode is still in its infancy, future research should investigate the impact of EAdi signal intensity on the prognosis of patients with cerebral hemorrhage. The aim of this study was to analyze whether the EAdi and EAdi-derived monitoring index were related to the prognosis of patients with severe cerebral hemorrhage.

## Methods

### Selected patients

Patients with cerebral hemorrhage and invasive mechanical ventilation who were admitted to the neurosurgery intensive care unit (NSICU) of Yijishan Hospital of Wannan Medical College from April 2019 to June 2021 were selected. The experimental protocol was approved by the institutional review board of Yijishan Hospital (No.51 of 2019). The trial was registered before patient enrollment at www.chictr.org.cn (primary registries of the WHO Registry Network, Clinical Trial Registration No. ChiCTR1900022861, principal investigator: Tao Yu, date of registration: April 28, 2019). Patients were eligible for inclusion based on the following criteria: (1) patients with cerebral hemorrhage, including those with subarachnoid hemorrhage (SAH) who received surgery and invasive mechanical ventilation; (2) endogenous positive end-expiratory pressure (PEEP) ≥ 3 cmH_2_O (1 cmH_2_O = 0.098 kPa); (3) informed consent signed by the patient or family members; and (4) administration of only short-acting sedative agents (i.e., propofol and/or sufentanil). Exclusion criteria were (1) pregnant or lactating women; (2) patients younger than 18-year old; (3) patients with cerebral hemorrhage not definitively diagnosed (such as hematological disorders); (4) patients receiving mechanical ventilation for less than 72 h; (5) hospice patients; (6) esophageal obstruction, esophageal perforation, severe esophageal varices, diaphragmatic hernia, and thoracic deformity; (7) severe heart, liver, kidney, and other organ failure (patients with expected short-term death); (8) severe coagulation dysfunction; (9) patients with severe respiratory central depression, high paraplegia, neuro-muscular disease; (10) patients with severe hemodynamic instability (MAP ≤ 65mmhg).

### Implantation of EAdi catheter and monitoring of EAdi signal

Each patient was consecutively ventilated for 72 h using a Servo-i ventilator (Getinge Group, Gothenburg, Sweden). A suitable EAdi catheter was selected according to the patient's height and weight. After the catheter was selected, the distance between the nose tip, earlobe, and xiphoid process of the patient was measured, and the approximate depth of EAdi catheter insertion was calculated. The EAdi was obtained using a catheter through a nasogastric tube with electrodes placed at its distal end. The study patients were kept moderately sedated (Propofol 600 mg and sufentanil 100ug + 48 ml normal saline were pumped intravenously at 2 mL/h).

### Data collection

#### General information record

Participants: Sex, age, height, weight, blood pressure, location of cerebral hemorrhage, Glasgow Coma Scale (GCS) score, Acute Physiology and Chronic Health Evaluation (APACHE) II score at ICU admission, past history, complications, ICU stay time, and GOS score of patients 6 months after discharge.

#### Ventilator-related indices

**Fig. 1:**
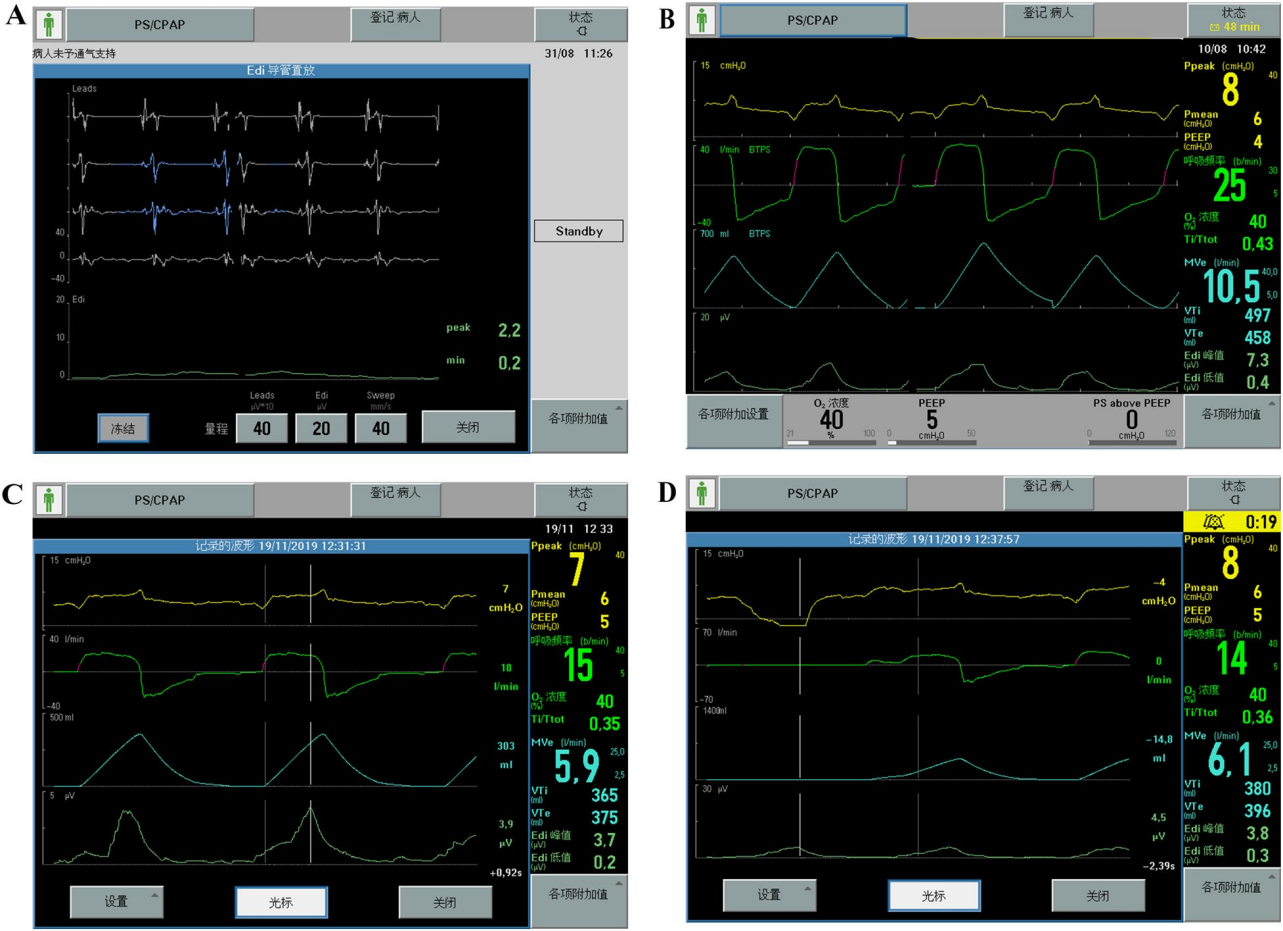
Study methods. **A** Confirmation of the EAdi catheter position; **B** measurement of the peak EAdi (EAdi_max_); **C** measurement of NVE, calculated as VT/EAdi_max_. **D** measurement of NME, calculated as (positive end-expiratory pressure − minimal airway pressure)/EAdi_max_. EAdi, electrical activity of the diaphragm; NVE, neuro-ventilatory efficiency; NME, neuro-muscular efficiency; VT, Tidal Volume

Mean airway pressure (Pmean), peak airway pressure (Ppeak), positive end-expiratory pressure (PEEP), tidal volume (VT), and minute ventilation (MV). The EAdi, EAdi-derived monitoring index, NVE, and NME were measured in the last 10 min of NAVA real-time PSV ventilation, in which each patient measured five groups of data once, took the average value, and measured for 3 consecutive days. NME was calculated as Paw-PEEP divided by EAdi during inspiratory occlusion. NVE was calculated as the ratio of VT to EAdi during inspiration. (Fig. 1).

### Statistical methods

SPSS version 21 (SPSS Inc., Chicago, IL, USA) was used for statistical analysis. The normally distributed data were described using mean numbers ± standard deviations, and the t-test of two independent samples was used for comparison between groups. Data for skewed distributions were represented by median (quartile) and the Wilcoxon nonparametric test was used for between-group comparisons. The use or composition ratio of counting data was described, and the χ2 test or Fisher exact probability method was used for comparison between groups. The difference was considered statistically significant at a *P* value < 0.05.

MedCalc (version 20, Ostend, Belgium) software was used to draw the receiver operating characteristic (ROC) curve and Kaplan–Meier curve. The ROC curve was drawn and the area under the ROC curve (AUC) was calculated. Kaplan–Meier survival curves were used to analyze the effects of EAdi, NVE, and NME on the 6-month survival. The Z test was used for comparison, and statistical significance was set at *p* < 0.05.

## Results

### Baseline characteristics

**Fig. 2:**
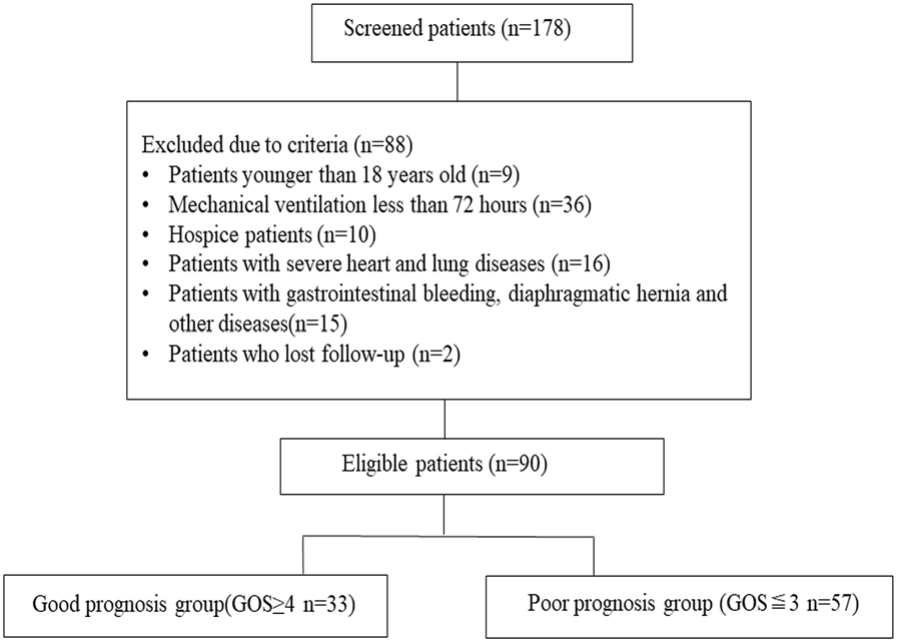
Flow chart of the study

A total of 178 patients were screened from April 2019 to June 2021. Finally, 90 patients were included in the study, of whom 33 were in the good prognosis group (GOS ≥ 4) and 57 were in the poor prognosis group (GOS ≤ 3), 12 patients (46%) died of respiratory failure, 6 patients (23%) died of intracranial hypertension, 5 patients (19%) died of infection, and 3 patients (12%) died of cardiovascular disease (Fig. 2). Patients with poor prognosis were older than those with good prognosis. The APACHE II score of patients with poor prognosis was significantly higher than that of patients with good prognosis (Table 1).

**Table 1:** Patients’ characteristics

Characteristics	GOS ≥ 4 (*n* = 33)	GOS≦3(*n* = 57)	*t/χ*^2^	*p*
*Clinical parameters*				
Age, years (mean ± SD)	53.03 ± 14.29	60.54 ± 12.89	**− **2.560	0.012
Sex, male/female, *n*	17/16	33/24	0.344	0.557
GCS score	7(5,10)	6(5,9)	1.055	0.304^a^
APACHE II	14.33 ± 2.62	16.14 ± 2.68	**− **3.130	0.003
BMI (kg/m^2^)	25.20 ± 3.21	24.64 ± 2.48	0.927	0.356
MAP, mmHg	88.42 ± 9.12	90.93 ± 9.16	**− **1.253	0.214
*Past history, n*			*χ*^2^	p
Cardiovascular disease	16	31	0.292	0.589
Cerebrovascular diseases	3	7	0.013	0.908
Endocrine diseases	3	3	–	0.665^*^
Kidney diseases	1	4	–	0.648^*^
Respiratory diseases	1	2	–	1.000^*^
*Comorbidities*			*χ*^2^	p
Pulmonary infection	23	30	2.514	0.113
Central system infection	5	19	3.533	0.060
Heart failure	1	6	–	0.416^*^
*Ventilatory parameters*			*χ*^2^	p
VT (ml)	558.309 ± 137.59	501.48 ± 118.60	2.348	0.021
MV (L)	9.43 ± 1.84	9.02 ± 2.06	1.196	0.274^a^
Pmean, cmH_2_O	7.49 ± 1.11	7.25 ± 1.23	0.943	0.348
Peep, cmH_2_O	5.28 ± 0.88	5.16 ± 0.95	1.188	0.276^a^
Ppeak, cmH_2_O	14.53 ± 3.15	13.43 ± 3.11	1.603	0.112
EAdi (µV)	4.7(3,7.7)	2.80(1.95,4.15)	11.850	0.001^a^
NVE (ml/µV)	141.85(90.52,212.06)	66.01(44.49,94.79)	21.100	< 0.001^a^
NME(cm H_2_O/µV)	2.57(1.71,4.75)	1.37(1.05,2.02)	13.828	< 0.001^a^
*Bleeding site, n*			*χ*^2^	*P*
CPH	18	36	0.646	0.422
SAH	14	17	1.469	0.225
IVH	1	4	–	0.648*

GOS, Glasgow Outcome Scale; GCS, Glasgow Coma Scale; APACHE II, Acute Physiology and Chronic Health Evaluation II; SOFA, Sequential Organ Failure Assessment; BMI, Body Mass Index; MAP, Mean Arterial Pressure; VT, Tidal Volume; MV, Minute Ventilation; Pmean, Mean Airway Pressure; PEEP, Positive End-Expiratory Pressure (PEEP); Ppeak, peak airway pressure; EAdi, electrical activity of the diaphragm; NVE, neuro-ventilatory efficiency; NME, neuro-muscular efficiency; CPH, Cerebral parenchymal hemorrhage; SAH, Subarachnoid hemorrhage; IVH, Intraventricular hemorrhage

Note: ^a^ Use the U test; * Use the Fisher exact probability method

### Comparison of ventilator-related index (Table 1)

1. There was no significant difference between the MV, Pmean, PEEP, and Ppeak in patients with good and poor prognosis.
2. The VT of patients with poor prognosis was lower than that of patients with good prognosis.
3. The EAdi, NVE, and NME in patients with good prognosis were significantly higher than those in patients with poor prognosis (4.70 µV vs 2.80 µV, *P* < 0.001; 141.85 ml/µV vs 66.01 ml/µV, *P* = 0.000; 2.57 cm H_2_O/µV vs 1.37 cm H_2_O/µV, *P* = 0.000).

### EAdi, NVE, and NME predictive value of patients’ survival prognosis (Table 2, Fig. 3)

**Table 2:** ROC data analysis in this study

	EAdi (µV)	NVE (ml/µV)	NME (cmH_2_O/µV)	EAdi + NVE + NME
Diagnostic accuracy (%)	68.89	75.56	75.56	85.56
Sensitivity (%)	69.70	75.76	69.70	84.85
Specificity (%)	68.42	75.44	78.95	85.97
Youden index	0.3812	0.5295	0.4864	0.7082
Positive predictive value (%)	56.10	64.10	65.71	77.78
Negative predictive value (%)	79.60	84.31	81.82	90.74
Cut-off value	3.6	95.32	2.06	–
AUC (95%*CI*)	0.719(0.614–0.808)	0.793(0.694–0.871)	0.792(0.694–0.871)	0.911(0.832–0.961)

**Fig. 3:**
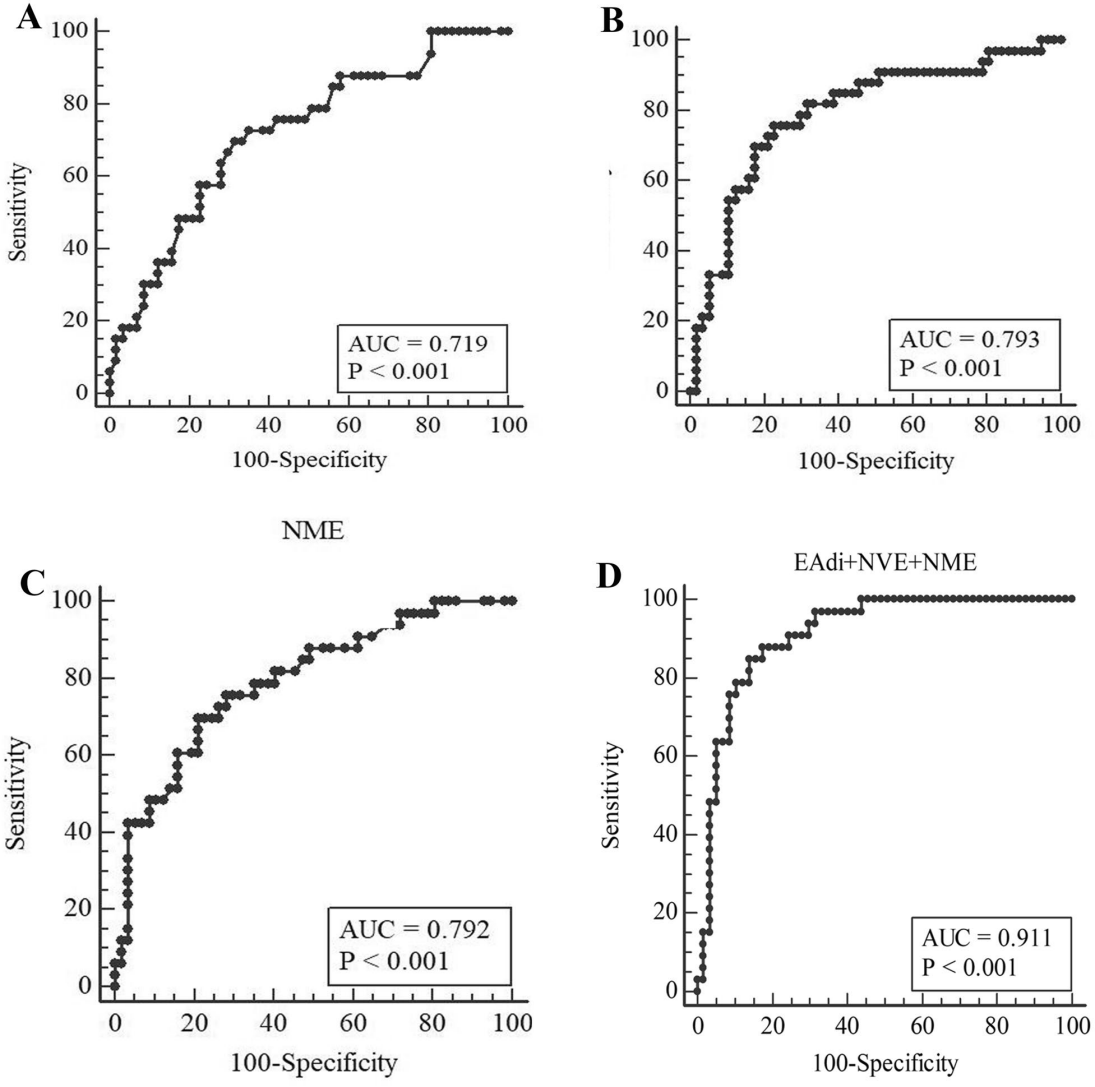
Upper panel shows the ROC curve for EAdi (**A**), NVE (**B**) and NME (**C**), the multi-variate (**D**) predicting the prognosis of patients with cerebral hemorrhage. The figure shows the ROC curve for EAdi predicting the prognosis of patients with intracerebral hemorrhage, having an AUC of 0.719 (*P* < 0.001). The ROC curve for NVE, having an AUC of 0.793 (*P* < 0.001). The ROC curve for NME, having an AUC of 0.792 (*P* < 0.001). The ROC curve for multi-variate (EAdi + NVE + NME), having an AUC of 0.911 (*P* < 0.001)

1. The AUC for the EAdi score was 0.719; when the EAdi was 3.6 µV, the sensitivity was 69.70%, and the specificity was 68.42%.
2. The AUC for the NVE score was 0.793; when the NVE value was 95.32 ml/µV, the sensitivity was 75.76%, and the specificity was 75.44%.
3. The AUC for the NME score was 0.792; when the NVE value was 2.06 H_2_O/µV, the sensitivity was 69.70%, and the specificity was 78.15%.
4. The AUC for the multi-variate (EAdi + NVE + NME) score was 0.911.

### Comparison of patient survival

**Fig. 4:**
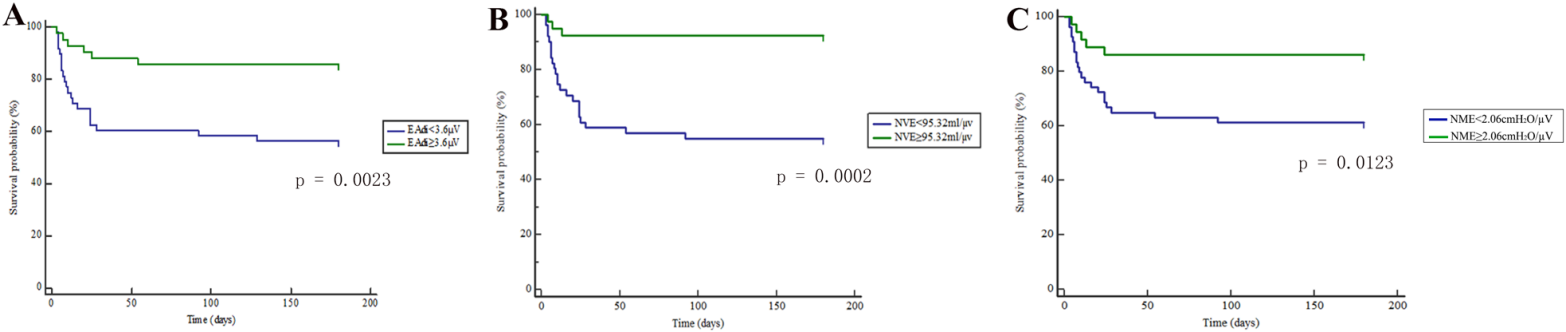
Kaplan–Meier curve was used to compare the 6-month cumulative survival rate of patients with cerebral hemorrhage. Grouping according to cut-off value of ROC curve of EAdi (**A**), NVE (**B**), NME (**C**), *P* < 0.05 was considered statistically significant. ROC, receiver operating characteristic curve; AUC, area under the curve; EAdi: electrical activity of the diaphragm; NVE, neuro-ventilatory efficiency; NME, neuro-muscular efficiency

The 6-month survival rate of patients with higher EAdi, NVE, and NME was significantly higher than that of patients with lower EAdi, NVE, and NME (Fig. 4).

## Discussion

Critical illness in patients is usually accompanied by direct or indirect respiratory or neuro-transduct damage, which is directly manifested as a lack or loss of the central drive. Studies have shown that many patients with severe disease have diaphragm dysfunction in the early stage of mechanical ventilation, and the long-term mortality and readmission rate of patients with diaphragm dysfunction at discharge are significantly higher than those of patients without diaphragm dysfunction [13, 14].

The EAdi signal is a new and unique ventilator monitoring parameter that provides clues for clinical diagnosis, and can be used as a decisive index to guide the ventilator support level [11, 15]. The EAdi is the sum of action potentials of diaphragmatic muscle fibers and is therefore the best index to reflect the central drive [16]. The EAdi reflects the neural respiratory drive, is the closest indicator that reflects the driving strength of the respiratory center, and is approximately 10 μV when normal healthy people are at rest. In our study, the EAdi signal of most patients with cerebral hemorrhage is lower than that of healthy people, possibly due to damage to the respiratory center or diaphragm function. The NVE describes the ability of the respiratory muscles to convert EAdi to tidal volume (VT/EAdi, ml/µV). The NVE is used to describe the ability of respiratory muscles to convert EAdi into ventilation volume, assess the proportion of respiratory muscle unloading, and predict the readiness for extubation [17, 18]. Studies have shown that NVE is a potential tool for predicting extubation readiness [19]. The NME is defined as delta airway pressure divided by EAdi measured during an end-expiratory occlusion and has been used to estimate inspiratory effort breath by breath [20–22].

Mechanical ventilation depends on the interaction of three key factors: central respiratory drive, respiratory muscle strength, and the load exerted on the respiratory muscle [23], and the EAdi is the best indicator of the central driving force [24, 25]. The strength of EAdi is an index of respiratory load, and it can be considered as an early warning index for reintubation or noninvasive ventilation [26]. When the patient's condition is improved, the decrease in EAdi signal intensity leads to a decrease in ventilator support level, which is an important indicator when considering weaning and extubation. In this study, the EAdi in patients with good prognosis was significantly higher than that in patients with poor prognosis, two patients had no EAdi and could not convert to NAVA, which was caused by inhibition of the respiratory center or damage to the nerve conduction pathway. In addition, the respiratory rate of two patients with brainstem hemorrhage in NAVA mode was accelerated, which was also due to the inhibition of the respiratory center and the decrease in EAdi, resulting in insufficient tidal volume and compensatory respiratory acceleration. This study also found that EAdi in patients with good prognosis tended to change at any time and gradually increased to the normal range, which was directly related to the gradual recovery of brain function, especially life center function. The EAdi signal is the intensity of the central nervous system impulse of the phrenic nerve, which plays an important role in the pathogenesis of acute respiratory failure; in this study, nearly half of the causes of death in patients with poor prognosis were respiratory failure.

The ROC curve analysis in this study showed that EAdi had a good predictive value for the prognosis of patients with cerebral hemorrhage (AUC = 0.719). When EAdi = 3.60 μV was the best cut-off value, the sensitivity and specificity for predicting the patient prognosis level were 69.70% and 68.42%, respectively, which showed that EAdi was a good predictive index. However, there are inter-individual and intra-individual variabilities in the EAdi values since it depends on underlying disease conditions, such as the presence of COPD, location (e.g., lobar, deep, or brainstem) and amount of ICH, Hunt and Hess grade for SAH, etc., and the EAdi signal is also affected by sedatives, so comparing the EAdi values between individuals may be meaningless. Therefore, we further studied the effect of NVE and NME on the prognosis of patients with cerebral hemorrhage, which reflects the ability of the diaphragm to convert the respiratory drive into ventilation.

The clinical relevance of the NVE index is that an increase in the index over time indicates that a patient can generate more VT for a given respiratory drive. The ROC curve analysis of our study showed that NVE had a better predictive value for the prognosis of patients with cerebral hemorrhage (AUC = 0.793). When NVE = 95.32 ml/μV was the best cut-off value, the sensitivity and specificity for predicting the patient prognosis level were 75.76% and 75.44%, respectively, which proved that NVE was a good predictive index. The low NVE value and patient's heavier condition can indicate that the patient's ventilation load is greater, and higher support may be needed at this time.

The NME describes how much pressure can be generated for each microvolt of the EAdi signal and the efficiency of the diaphragm in generating pressure for a certain amount of electrical activity. The NME outlines the ability to convert EAdi to inspiratory pressure. The NME helps to titrate ventilatory support to minimize diaphragm dysfunction resulting from ventilator over-assist and under-assist [27]. In the present study, we found that NME was significantly lower in patients with poor prognosis, indicating a weaker diaphragm. When NME = 2.06 cmH_2_O/μV was the best cut-off value, the sensitivity and specificity for predicting the patient prognosis level were 69.70% and 78.95%, respectively, which proved that NME was a good predictive index (AUC = 0.792).

EAdi is the best indicator of the central driving force, and the diaphragm is the most important respiratory muscle in the human body, and its contraction is completed under the control of the respiratory center. If the patient's central nervous system or respiratory center is damaged, their diaphragm function may be affected, the EAdi is directly related to the respiratory center, so monitoring EAdi, NVE, and NME can evaluate the respiratory drive of the respiratory center to the diaphragm. Phrenic nerve stimulation may achieve lung and diaphragm protection at the same time. Ahn studied unilateral phrenic nerve stimulation during cardiac surgery and found that stimulation every 30 min can increase diaphragm contractility by 30% compared with the healthy side [28]. For patients with intracerebral hemorrhage, early phrenic nerve stimulation may improve the prognosis of patients. By monitoring patients' EAdi, NVE and NME, we can intuitively evaluate the diaphragm function of patients.

In future studies, it is often necessary to further expand the sample size and adopt a multicenter randomized controlled study to compensate for study deficiencies. In addition, because this was a single-center retrospective study, the sample size was small, dynamics were lacking, and the applicable population was limited, we cannot completely eliminate the interference of other factors, and it is difficult to characterize the target study population because of the broad inclusion criteria (ICH, SAH, and IVH, etc.) and not meticulously collecting variables regarding patients’ condition (for example, location (e.g., lobar, deep, or brainstem) and amount of ICH, Hunt and Hess grade for SAH, etc.). It should be pointed out that the patients in this study were all severe cerebral hemorrhage and could not represent all patients with cerebral hemorrhage. In the future, we will expand the sample size and adopt a multicenter and prospective research approach to further analyze the relationship between EAdi, NVE, and NME, in postoperative patients with cerebral hemorrhage.

## Conclusion

Patients with a good prognosis have increased EAdi, reflecting a higher neural respiratory drive, and increased NME and NVE indicating more pronounced respiratory muscle strength. The EAdi curve and EAdi-derived monitoring index have predictive value for patient prognosis.

## Data Availability

The data sets used and/or analysed during the current study are available from the corresponding author on reasonable request.
